# Frailty in Community-Dwelling Older People

**DOI:** 10.3390/healthcare11162298

**Published:** 2023-08-15

**Authors:** Robbert J. Gobbens

**Affiliations:** 1 Faculty of Health, Sports and Social Work, Inholland University of Applied Sciences, 1081 HV Amsterdam, The Netherlands; robbert.gobbens@inholland.nl; 2 Zonnehuisgroep Amstelland, 1186 AA Amstelveen, The Netherlands; 3 Department Family Medicine and Population Health, Faculty of Medicine and Health Sciences, University of Antwerp, 2610 Antwerp, Belgium; 4 Department of Tranzo Academic Centre for Transformation in Care and Welfare, Faculty of Behavioural and Social Sciences, Tilburg University, 5037 AB Tilburg, The Netherlands

With a growing aging population around the world [1], frailty is becoming an increasingly important topic as it is closely related to older ages [2]. To deal with the challenges resulting from the aging population, services and policies are increasingly focused on independent living in the community rather than relying on institutions, e.g., nursing homes. This fits in the wish of many older people, who prefer to stay in their own homes for as long as possible [3]. However, frail older people living independently in the community have a high risk for a lower quality of life [4], disability [5], an increase in healthcare utilization (e.g., hospitalization, institutionalization) [5,6], and mortality [7]. Therefore, it is important that we gain more knowledge about the identification and assessment of frailty, determinants of frailty, the associations between frailty and adverse outcomes, perspectives of healthcare professionals on frailty (e.g., general practitioners, nurses), and most importantly, how to prevent or delay frailty.

This Special Issue contains fourteen papers. Three studies were conducted in Japan. The study by Tsujishita et al. [8] aimed to investigate whether the overlap of physical, cognitive, and social frailty affected Ikigai in community-dwelling older people (mean age 75.1 ± 5.7 years). Ikigai refers to the satisfaction with social relationships in addition to personal satisfaction and happiness. Fujii et al. [9] performed a cross-sectional study with the aim to clarify the association between frailty/occupational dysfunction and well-being in a sample of 2308 community-dwelling older individuals. The third Japanese study was carried out by Yokote et al. [10] to determine whether older people who walk for exercise have a lower risk of physical frailty than those who do not. To assess physical frailty, Yokote et al. utilized the frequently used Cardiovascular Health Study criteria by Fried et al. [11]. 

“How to Make Primary Healthcare More Popular” is the title of the study by Fu et al. [12]. These researchers discovered that the supply of primary health services had a significant direct and mediating effect on the utilization of primary outpatient services in China. In the same country, Li et al. [13] found that intestinal permeability was associated with the loss of skeletal muscle strength in middle-aged and older adults. According to Li et al., serum diamine oxidase may be a novel predictor for the loss of skeletal muscle strength in middle-aged and older males.

Tasioudi et al. [14] identified the effect of frailty and geriatric syndromes, including comorbidities, on the quality of life of Greek people aged 65 years or older receiving home care. A second Greek study was conducted by Aravantinou-Karlatou et al. [15]. They evaluated frailty and its association with geriatric syndromes (cognitive function, depression, and comorbidities) in the context of socioeconomic variables (e.g., education and annual income).

This Special Issue also includes a systematic review and meta-analysis by To et al. [16] who aimed to synthesize frailty prevalence among community-dwelling older people in Asia and to identify factors influencing prevalence estimates. The study provides references for health policy decision-making regarding the prevention of frailty progression in Asian countries. Alshanberi [17] also pays attention to the prevalence of frailty. In his paper, he presents studies on frailty focused on older people living in the Kingdom of Saudi Arabia.

Two papers describe studies conducted in the Netherlands. One of these studies examined the opinions of nurses on frailty (Gobbens et al.) [18]. The nurses mainly associated frailty with physical deterioration and dementia, but the social and psychological domains were also often mentioned, pointing to a holistic approach to frailty. The second study by Gobbens et al. [19] was focused on the prediction of disability in (instrumental) activities of daily living using a nomogram of the Tilburg Frailty Indicator (TFI), a user-friendly self-report questionnaire [20].

The deterioration of cognitive and psychophysical ability associated with aging has an effect on road safety, especially in the driving of vehicles. Mirabet et al. [21] evaluated the psychophysical attitudes in drivers over 65 years in Spain. The effect of the active aging-in-place rehabilitation nursing program on the functional capacity and lifestyle of frail Portuguese older people was examined using a randomized controlled trial by Faria et al. [22]. Finally, this Special Issue also contains an observational, longitudinal, cohort study by Gentili et al. [23] aimed at evaluating the admission rate to long-term care facilities for community-dwelling older people. In addition, this study examined the factors associated with these admissions.

In conclusion, this Special Issue offers a wide range of papers that all contribute to our knowledge about frailty in older people. This knowledge is much needed given the increasing global number of frail community-dwelling older people and helps ensure that their quality of life is not diminished.
